# Infected Concha Bullosa Mucocele: A Case Report

**DOI:** 10.7759/cureus.22538

**Published:** 2022-02-23

**Authors:** Dakheelallah Almutairi, Ashwaq Alosaimi, Atheer Alsurayhi, Atheer Altalhi

**Affiliations:** 1 Otolaryngology - Head and Neck Surgery, College of Medicine, King Saud bin Abdulaziz University for Health Sciences, Jeddah, SAU; 2 Otolaryngology - Head and Neck Surgery, King Abdullah International Medical Research Center, Jeddah, SAU; 3 Otolaryngology - Head and Neck Surgery, King Abdulaziz Medical City, Ministry of National Guard Health Affairs, Jeddah, SAU; 4 College of Medicine, Umm Al-Qura University, Makkah, SAU; 5 Otolaryngology - Head and Neck Surgery, Dr. Soliman Fakeeh Hospital, Jeddah, SAU

**Keywords:** neoplasm, intranasal mass, mucocele, unilateral nasal obstruction, concha bullosa

## Abstract

Concha bullosa (CB) is a common sinonasal anatomic variant. The obstruction of a CB, though rare, might result in mucocele that may be misdiagnosed. In this report, we present a case of a 32-year-old female with a one-year history of unilateral nasal obstruction, headache, facial pain, foul nasal discharge, and hyposmia, initially misdiagnosed as a neoplasm. Computed tomography (CT) and magnetic resonance imaging (MRI) revealed a right middle CB infection with a mucocele. Laboratory cultures yielded *Pseudomonas aeruginosa*. Endoscopic sinus surgical mass excision was performed, and treatment with oral cefuroxime was administered. The patient recovered fully. A CB mucocele, though rare, should be considered in the differential diagnosis of an intranasal mass.

## Introduction

Concha bullosa (CB) is a unilateral or bilateral pneumatization of the nasal conchae and a common sinonasal anatomical variant with prevalence rates of 14%-53% [1]. CB is often asymptomatic and is mostly an incidental finding on computed tomography (CT) [2]. CB occurs when a pneumatic expansion from anterior ethmoid cells or less usually from posterior cells invades the middle concha of the nasal cavity. Generally, a CB has only one air cell, and many air cells within a CB are unusual [1]. CB is categorized according to the anatomical location. It is termed lamellar if the pneumatization is at the vertical lamella level, bulbous if it is at the inferior bulbous level, and extensive if it involves the entire concha [2]. The mucociliary transport system of the CB empties into the frontal recess or the middle meatus through the sinus lateralis. The obstruction of a CB might result in a mucocele [3]. A mucocele is a mucus-filled sac with epithelial cell lining [4]. It is common in the ethmoid and/or frontal sinuses [4,5]. An infected mucocele is called a pyocele [5], and its occurrence in the CB is extremely rare [6]. In this case report, we report an unusual presentation of an infected CB with mucocele.

## Case presentation

A 32-year-old Saudi female was referred to King Abdulaziz Medical City in Jeddah (KAMC-J) as a case of inverted papilloma for further evaluation. The patient complained of right-sided nasal obstruction for a duration of one year and headache, facial pain, nasal discharge, and hyposmia. Endoscopic examination identified a right-side nasal polyp; it appeared to arise from the lateral nasal wall, and it occupied the middle meats, while the left side was clear and patent. CT scan done in the referring hospital revealed a nasal mass extending from the lateral nasal wall to the maxillary sinus, displacing the lamina papyracea. CT of the sinuses was not done in KAMC-J because the patient was pregnant at that time.

**Figure 1:**
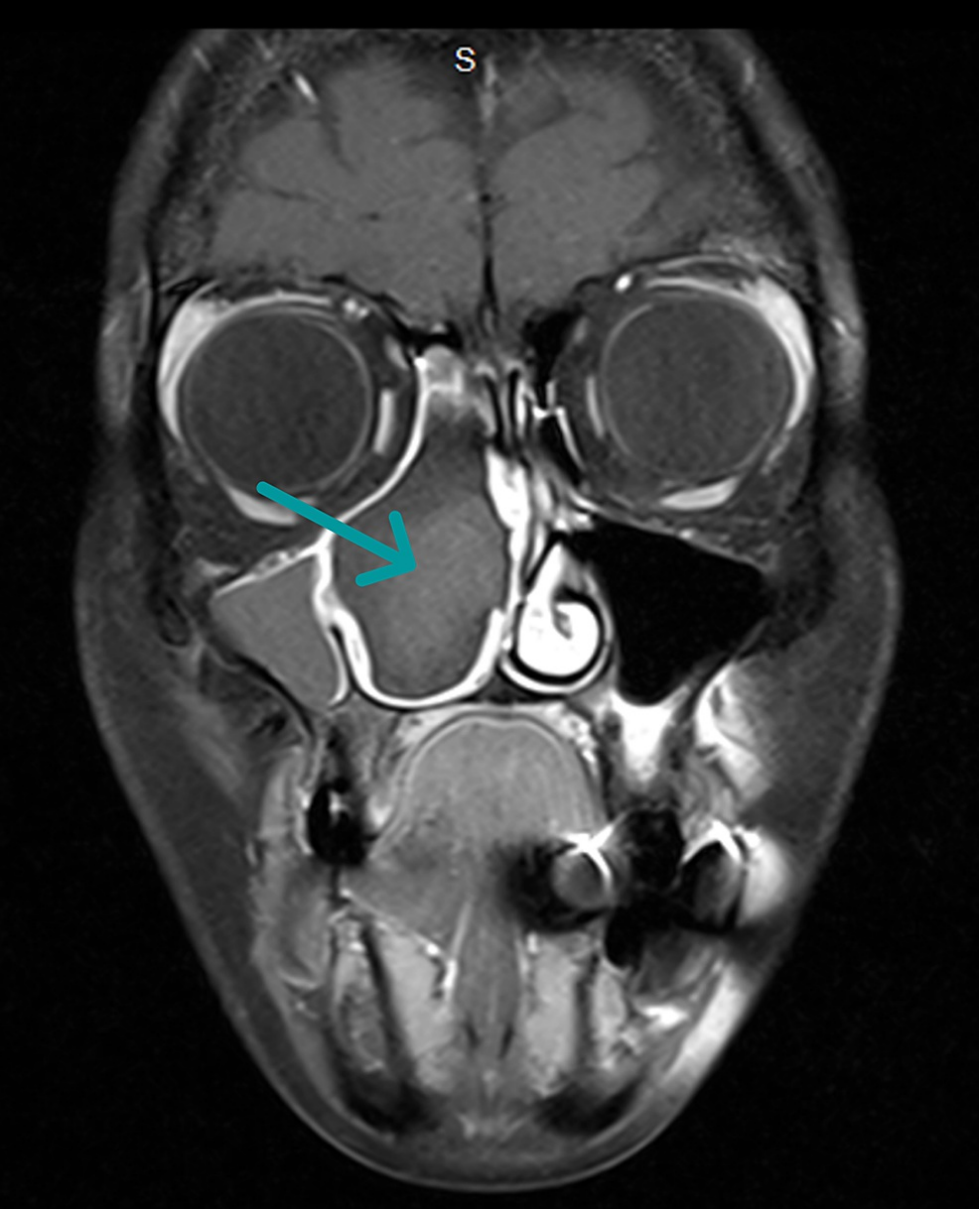
Brain and neck MRI The T1 coronal image shows a right maxillary sinus mass with iso-to-high signal intensity.

**Figure 2:**
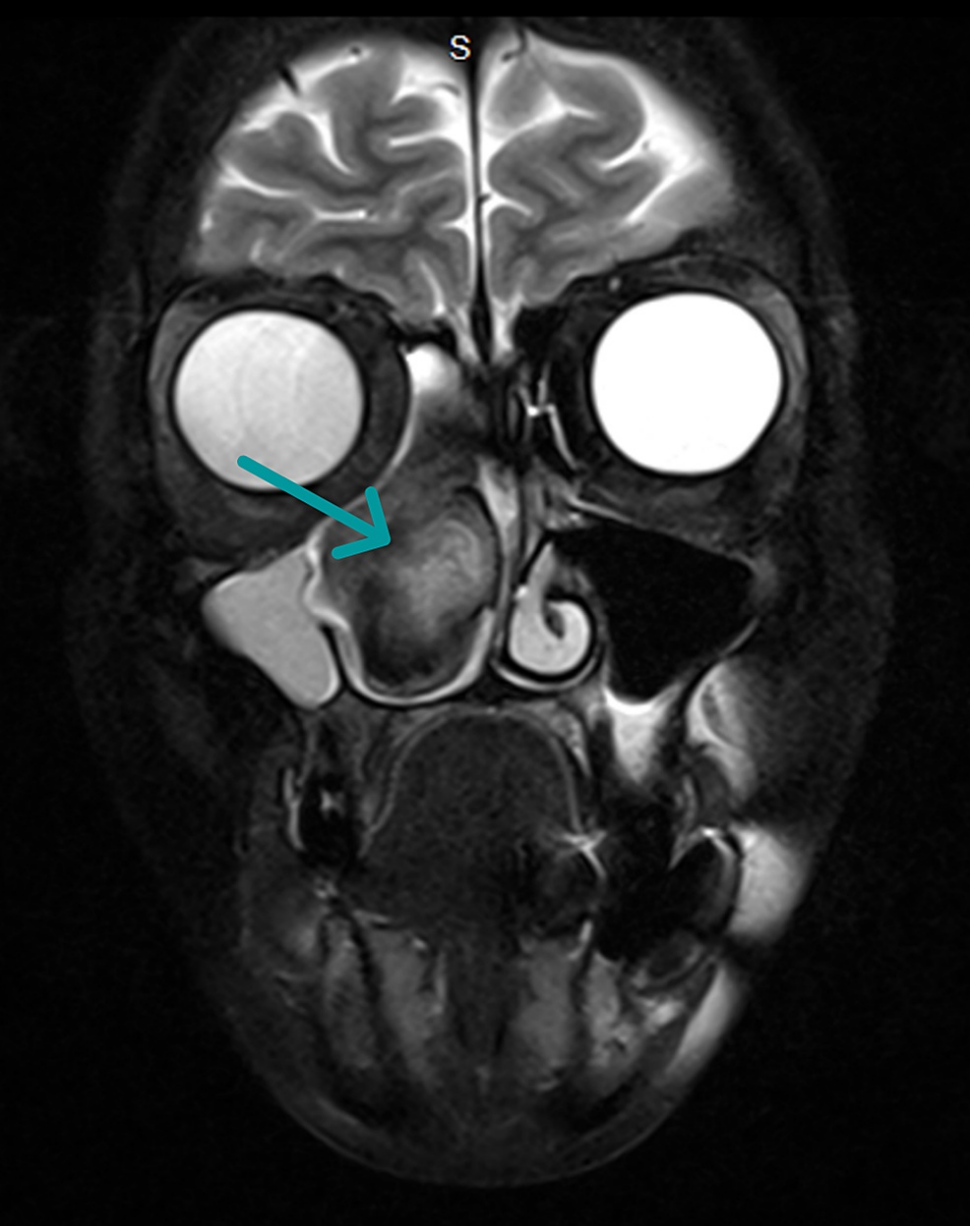
Brain and neck MRI The T2 coronal image shows a right maxillary sinus mass with heterogeneous signal intensity.

However, a biopsy of the right nasal mass was consistent with an inflammatory sinonasal polyp. After delivery, CT of the sinuses showed an expansile right nasal cavity mass with septal displacement to the left. Brain and neck magnetic resonance imaging (MRI) revealed a right maxillary sinus expansile mass with iso-to-high signal intensity in T1 and heterogeneous signal intensity in T2. The mass affected the adjacent structures, shifting the nasal septum to the left and the medial wall of the right maxillary sinus to the right, suggestive of a right middle CB infection with a mucocele (Figures 1, 2).

**Figure 3:**
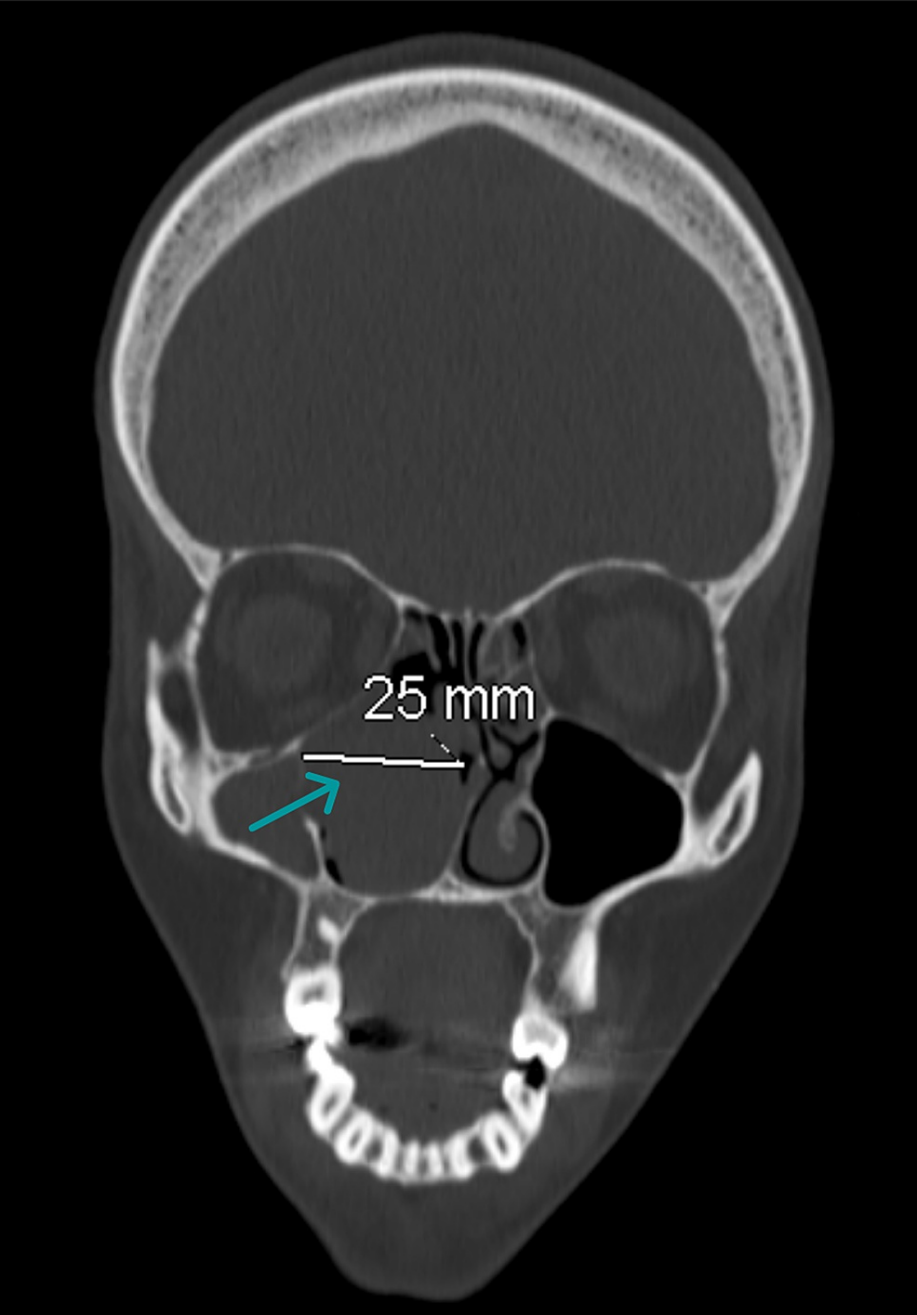
Preoperative CT of the paranasal sinus The coronal view shows the measurement of a concha bullosa mucocele that fills the right nasal cavity and an opposite nasal septum deviation.

**Figure 4:**
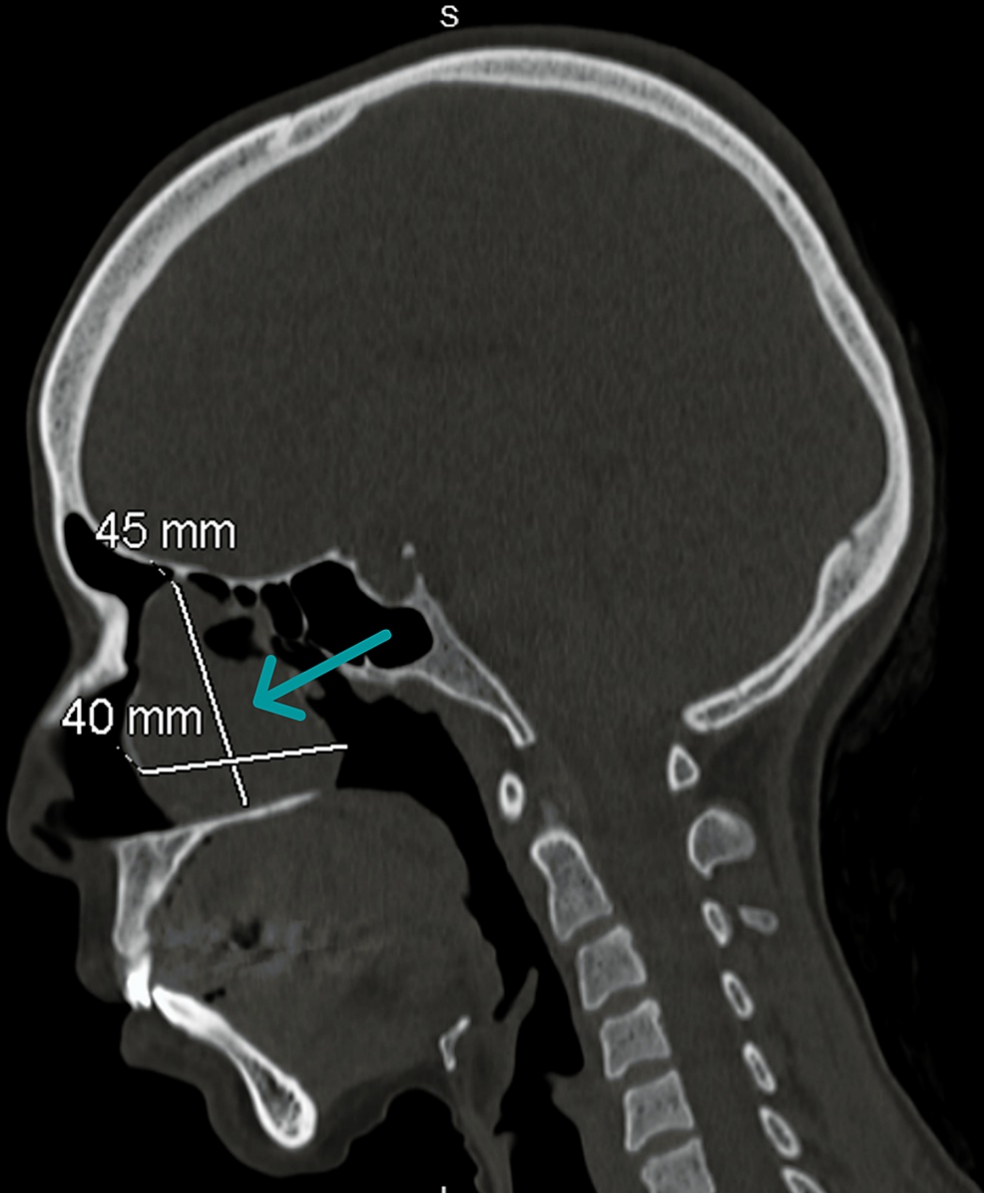
Preoperative CT of the paranasal sinus The sagittal view shows the measurement of a concha bullosa mucocele.

The patient was followed up in the otolaryngology outpatient clinic since March 2020; however, her symptoms progressed to epiphora, sensation of facial pressure, and blurred vision in the right eye. Endoscopic examination confirmed that a right nasal polyp obstructed the nasal cavity and a left laterally displaced nasal septum. Ophthalmologic examination of both eyes showed normal global positions, visual acuities of 20/20, normal intraocular pressures, ocular** motility, and pupillary examinations**. From the clinical and radiological findings, a diagnosis of right CB mucocele was established, and functional endoscopic sinus surgery (FESS) with septoplasty was scheduled. Preoperative CT of the sinuses revealed a mild interval reduction in the size of the right nasal cavity lesion (Figures 3, 4).

**Figure 5:**
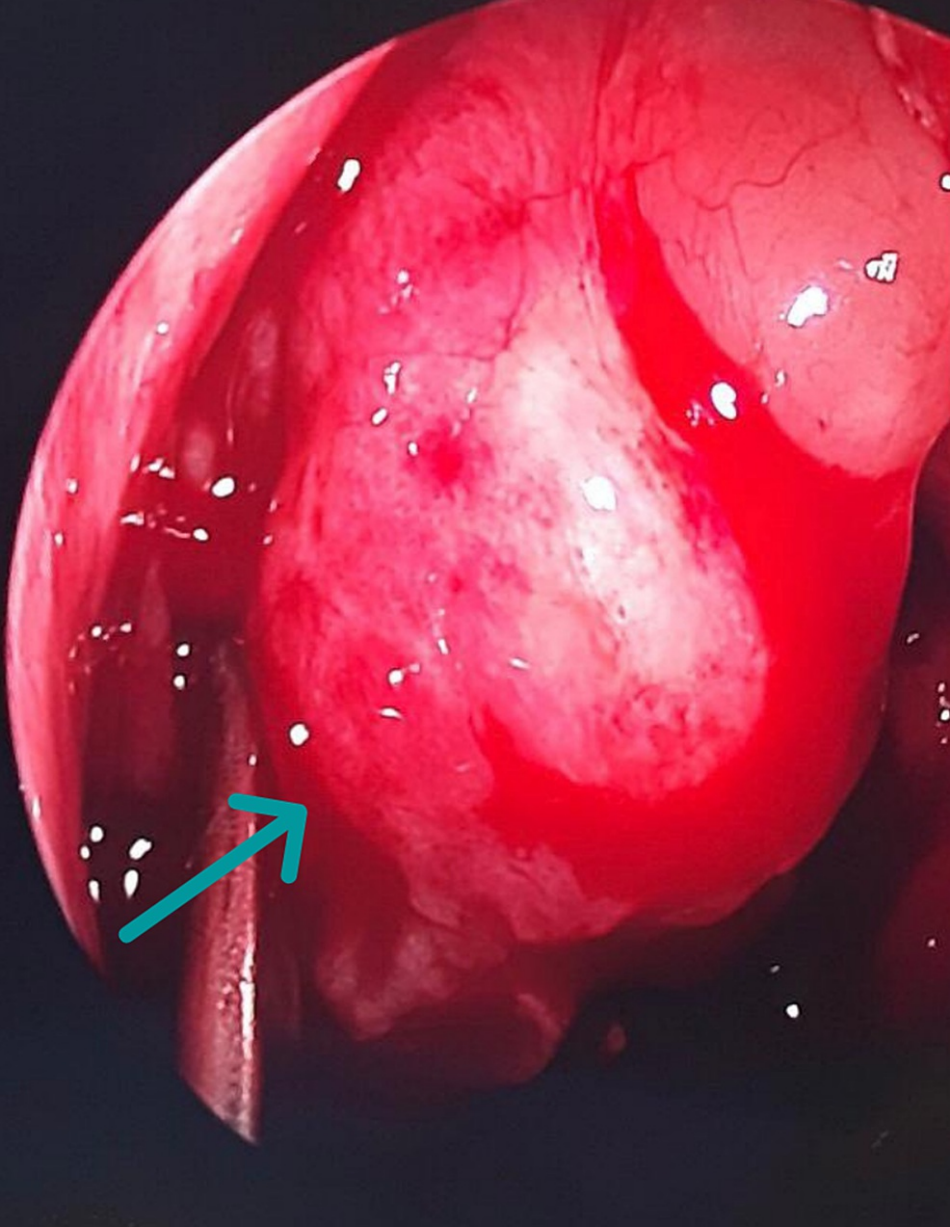
Intraoperative endoscopic view of the nasal polyp covered with smooth mucosa

Under general anesthesia, an endoscopic examination showed right nasal polyposis (Figure 5). Debridement was done, and the CB was full of purulent discharge. Two biopsies were taken for histopathological examination, and culture samples were sent for further evaluation. Then, the lateral part of the middle turbinate was resected. A right maxillary antrostomy was done to drain and ventilate the maxillary sinus. The left nasal cavity was examined, which was clear of any polyps or secretions, and endoscopic septoplasty was performed. Oral antibiotic treatment with cefuroxime, 500 mg twice daily, for 10 days was prescribed for a concomitant paranasal sinus infection.

Histopathology reported CB with mucoid material, fibrin, and admixed bacterial morphology. The culture was positive for *Pseudomonas aeruginosa* (*P. aeruginosa*). At the three-month post-operative follow-up visit, the patient had no symptoms, and nasal cavity examination revealed patent nasal passages with no synechiae.

## Discussion

This study presents a case of chronic sinusitis due to maxillary sinus obstruction and polyps in an adult. After an initial misdiagnosis, a definitive diagnosis of a CB mucocele infected with *P. aeruginosa* is made and treated by endoscopic surgery and antibiotics, followed by a full patient recovery. The patient’s presenting symptoms of right-sided nasal obstruction, headache, facial pain, nasal discharge, and hyposmia are the effects of an obstruction in the mucociliary transport system causing mucoceles of the paranasal sinuses or CB. This obstruction may occur from chronic rhinosinusitis, nasal polyps, facial trauma, or previous sinus surgery, resulting in the accumulation of secretions and an expanding mass [7], which impinges on the surrounding structures, causing the presenting symptoms [8].

Mucoceles are most common in the frontoethmoidal region, maxillary sinus, sphenoid sinus, or posterior ethmoid in adults, and in 90% of cases, they are unilateral. However, mucoceles (or pyoceles) are mostly seen in the paranasal sinus, not in the CB [9]. CB mucoceles may present with nasal obstruction, headache, nasal discharge, postnasal drip, hyposmia, orbital pain, and exophthalmia [3]. In the studied case, the mucocele caused nasal obstruction, nasal discharge, headache, and septal deviation.

CB mucocele may present as a large nasal mass surrounded by a thin bone plate and covered with intact mucosa. CB mucoceles have more surrounding space than the mucoceles within maxillary, ethmoid, or frontal sinuses, which allows them to expand to large sizes before being detected. This expansion displaces the septum to the other side and obliterates the maxillary, ethmoid, and frontal sinus. Anterior rhinoscopy may reveal a large mass filling nasal cavity that is covered with smooth intact respiratory mucosa. The diagnosis is based mainly on the radiological characteristics of CT and MRI scans.

Despite the presenting symptoms, the case was initially diagnosed as an inverted papilloma by CT; however, further evaluation by MRI reveals the final diagnosis of a CB mucocele. Because mucoceles may be misdiagnosed as tumors, this differential diagnosis should be considered in the assessment of an intranasal mass. The possibility of a polyp, papilloma, or tumor in a unilateral mass of the nasal cavity should be explored [8]. A diagnosis is made by cross-sectional imaging, endoscopy, and biopsy. A combined CT and MRI usually provide the most comprehensive information [7]. MRI can also distinguish between a mucocele and an expansile mass [9]. To our knowledge, this is an exceptional case of a CB mucocele misdiagnosed as a nasal neoplasm.

In this case report, the microbiological investigation of the infected CB reveals *P. aeruginosa*. This is consistent with reports in two previous cases in Turkey in 2013 [8]. However, in reported cases of infected mucoceles, the most common isolated organism was *Staphylococcus aureus* [10]. A CB mucocele is best treated with endoscopic surgery using any of the four methods: lateral marsupialization, medial marsupialization, crushing, or transverse excision [3]. In the reported case, lateral marsupialization was used.

## Conclusions

This case, an infected CB with a mucocele, is a rare condition that may occur without any predisposing factors. Therefore, a CB mucocele should be considered as a differential diagnosis of any slow-growing mass when a definitive diagnosis cannot be made without complementary imaging and endoscopic surgery, which are the main diagnostic and therapeutic management approaches in this case, respectively.
